# Green microalgae in marine coastal waters: The Ocean Sampling Day (OSD) dataset

**DOI:** 10.1038/s41598-018-32338-w

**Published:** 2018-09-19

**Authors:** Margot Tragin, Daniel Vaulot

**Affiliations:** Sorbonne Université, CNRS, UMR 7144, Station Biologique, Place Georges Teissier, 29680 Roscoff, France

## Abstract

The ecology and distribution of green phytoplankton (Chlorophyta) in the ocean is poorly known because most studies have focused on groups with large cell size such as diatoms or dinoflagellates that are easily recognized by traditional techniques such as microscopy. The Ocean Sampling Day (OSD) project sampled surface waters quasi-simultaneously at 141 marine locations, mostly in coastal waters. The analysis of the 18S V4 region OSD metabarcoding dataset reveals that Chlorophyta are ubiquitous and can be locally dominant in coastal waters. Chlorophyta represented 29% of the global photosynthetic reads (Dinoflagellates excluded) and their contribution was especially high at oligotrophic stations (up to 94%) and along the European Atlantic coast. Mamiellophyceae dominated most coastal stations. At some coastal stations, they were replaced by Chlorodendrophyceae, Ulvophyceae, Trebouxiophyceae or Chlorophyceae as the dominating group, while oligotrophic stations were dominated either by Chloropicophyceae or the uncultured prasinophytes clade IX. Several Chlorophyta classes showed preferences in terms of nitrate concentration, distance to the coast, temperature and salinity. For example, Chlorophyceae preferred cold and low salinity coastal waters, and prasinophytes clade IX warm, high salinity, oligotrophic oceanic waters.

## Introduction

Marine waters are inhabited by a heterogeneous assemblage of organisms that includes a large diversity of unicellular eukaryotes (protists). Photosynthetic protists (phytoplankton) are responsible for the bulk marine primary production. Photosynthetic organisms are divided into two lineages, green and red. The former originates from primary endosymbiosis and includes Chlorophyta^1^, the major green algal group in marine waters, as well as vascular plants. The latter has undergone secondary and even tertiary endosymbioses and is represented among other by diatoms and dinoflagellates that are key photosynthetic producers in productive marine waters. Chlorophyta have a chloroplast surrounded by only two membranes and possess chlorophyll *b* as the main accessory chlorophyll. The Chlorophyta division is composed of two major groups: the prasinophytes and the “core” Chlorophytes^2,3^. The “core” Chlorophytes consist of Ulvophyceae, Trebouxiophyceae and Chlorophyceae (known as UTC clade) to which two classes Pedinophyceae and Chlorodendrophyceae have been recently added^2^. Prasinophytes consist currently of eight major lineages of microalgae corresponding to different taxonomic levels (Order, Class, undescribed clades). The number of prasinophyte lineages has been increasing following the availability of novel cultures and environmental sequences. More than a decade ago, prasinophyte clade VII was added in order to regroup sequences from cultured strains and environmental clone libraries^4^. Four years later, two additional clades, VIII and IX, were reported^5^ that are only known so far from environmental sequences. In recent years, some of the prasinophyte clades have been raised to the Class level. As an example, Leliaert *et al*.^6^ used multigenic phylogenies to establish a new class, the Palmophyllophyceae, which gathers the orders Prasinococcales and Palmophyllales. Clade VII has been recently split into 2 new classes, Chloropicophyceae and Picocystophyceae^7^.

The ecology and distribution of green phytoplankton in the ocean is poorly known since most studies have focused on groups that are easily identified by microscopy and cause massive blooms such as diatoms or dinoflagellates, leading to the view that ocean is dominated by the red lineage^8^. Representatives of green algae are mostly found in small size fractions, in particular the picophytoplankton (cells from 0.2 to 2 µm) and nanophytoplankton (cells from 2 to 20 µm), which are key primary producers in central oceanic regions^9^. Differences in the distribution of major classes or clades have already been demonstrated between coastal and oceanic waters. Mamiellophyceae are the major Chlorophyta contributors in coastal water, while Chloropicophyceae^10^ and prasinophytes clade IX^11^ dominate oceanic waters. However, no global analysis of the relative importance and distribution of the different green algal groups in the ocean has yet been performed.

High Throughput Sequencing (HTS) methods provide large metabarcoding datasets which enable the exploration of the diversity and distribution of protist groups in the ocean^12^. The Ocean Sampling Day (OSD) project^13^ sampled in 2014 the global ocean, mostly at coastal stations, at the boreal summer solstice (June 21). At each station, the V4 region of the 18S rRNA gene was sequenced. In this paper, we analyze the OSD V4 metabarcoding datasets with the aim to describe the distribution of the major classes of Chlorophyta in the global coastal ocean.

## Materials and Methods

### Sampling and sequencing

157 water samples from 145 marine locations were filtered on 0.22 µm pore size Sterivex without prefiltration and frozen at −80 °C. Metadata (Temperature, Salinity, Nutrients and Chlorophyll *a*) were obtained from https://github.com/MicroB3-IS/osd-analysis/wiki/Guide-to-OSD-2014-data. Temperature and salinity were measured *in situ* during the sampling. Nutrients concentration were estimated from historical data uploaded from the World Ocean Database 2013^14^ (https://www.nodc.noaa.gov/OC5/WOD13/) and Chlorophyll *a* was estimated from remote sensing ocean color from the MODIS AQUA database (Moderate Resolution Imaging Spectroradiometer, http://oceancolor.gsfc.nasa.gov/cgi/l3). In this paper, we only considered 141 samples obtained from the surface layer (Table S1).

All molecular processing steps were performed by the OSD team. DNA was extracted using the Power Water isolation kit (MoBio, Carlsbad, CA, USA) following the manufacturer instructions. V4 was amplified using TAReuk454FWD1 (5′-CCA GCA SCY GCG GTA ATT CC-3′) as forward primer and the modified TAReukREV3_modified (5′-ACT TTC GTT CTT GAT YRA TGA-3′) as reverse primer^15,16^. The Illumina libraries were prepared using the Ovation Rapid DR Multiplex System 1–96 (NuGEN, link to protocol:https://owncloud.mpi-bremen.de/index.php/s/RDB4Jo0PAayg3qx?path=/2014/protocols). Sequencing (2 × 250 paired end) was done with Illumina technology MiSeq using V3 chemistry by the LGC genomics GmbH (Germany, http://www.lgcgroup.com/).

### Data processing

R1 and R2 were filtered based on quality and length and assembled by the OSD consortium which provided the so-called “workable” fasta files (http://mb3is.megx.net/osd-files?path=/2014/datasets/workable). This dataset provided around 5 million workable V4 region of the 18S rRNA gene metabarcodes. The raw files have been deposited at EBI (http://www.ebi.ac.uk/ena/data/view/ERX947554).

All subsequent sequence processing was done with Mothur v 1.35.1^17^. Reads were filtered to remove those shorter than 300 bp or containing ambiguities (N). Then, reads were aligned to SILVA seed release 123 alignment^18^ corrected manually with the Geneious software v 7.1.7^19^: gaps at the beginning and the end of sequences were deleted. The aligned datasets were filtered by removing columns containing only insertions. Chimeras were checked using Uchime v 4.2.40^20^ as implemented in Mothur. The datasets were pre-clustered using Mothur. After distance matrix calculation, the sequences were clustered using the Nearest Neighbor method and Operational Taxonomic Units (OTUs) were built at 99% similarity. OTUs represented by only one sequence (singletons) were deleted. OTUs were finally assigned using the Wang approach^21^ and the PR² database^22^, available at 10.6084/m9.figshare.5913181, for which the Chlorophyta sequences had been checked against the most recent taxonomy^23^. OTUs for which assignation bootstrap value was lower than 80% were not taken into account. Each OTU was linked to a reference sequence and an OTU was considered to be assigned when the lowest taxonomic level (“Species” level in PR^2^) differed from “unclassified”. In order to validate OTU assignation, all OTU reference sequences were further BLASTed against the GenBank nr database using megablast.

### Statistical analyses

**Figure 1:**
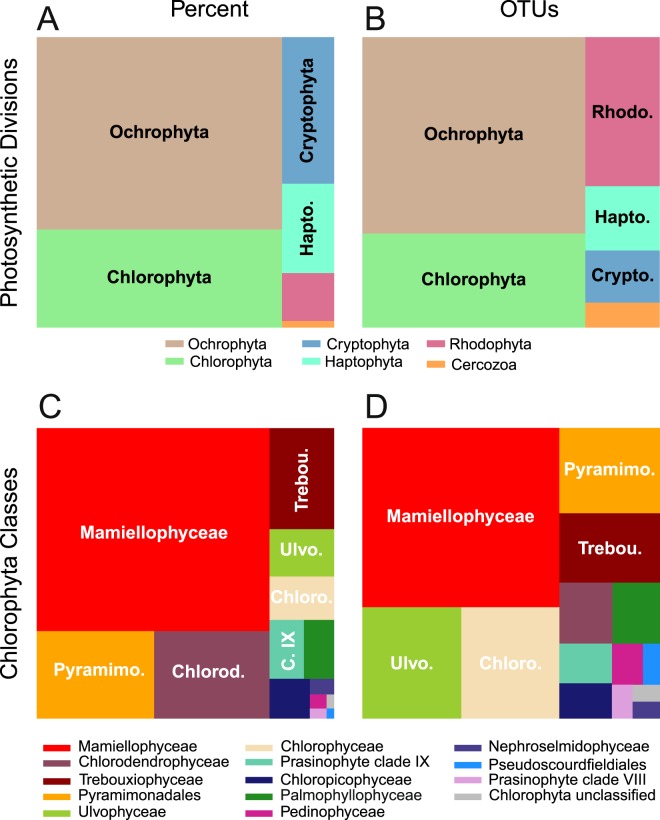
Contribution (average number of reads per station) and diversity (number of OTUs) of photosynthetic group at OSD stations. (**A**) Photosynthetic divisions (Total number of reads = 1,103,675). (**B**) Idem for OTUs (Total = 3069). (**C**) Chlorophyta classes (Total number of reads = 313,240). (**D**) Idem for OTUs (Total = 749).

For all analyses of the relative abundance of specific classes, we only considered samples for which more than 100 Chlorophyta reads were obtained (122 samples). Graphics and ecological analyses were performed using the R v. 3.0.2 software (http://www.R-project.org/). We used the package Treemap to draw treemaps (Fig. 1), ggplot2^24^ to draw maps and bar graphs, ComplexHeatmaps^25^ for heatmaps, Gplots for all other plots. Distance to the coast was calculated for each station using Rgdal and Rgeos packages and the coastline file available (http://www.naturalearthdata.com/downloads/10m-physical-vectors/10m-coastline/). The Vegan package^26^ was used to compute slopes of rarefaction curves (function *rareslope*) and Bray-Curtis dissimilarity matrices (function *vegdist*).

## Results

### The OSD sampling network

All OSD stations (Table S1) were sampled around the same date, June 21, 2014, the boreal summer solstice. In contrast to other global surveys such has Tara *Oceans*^27^, OSD stations were mostly coastal: distance from the coast varied from a few meters to more than 300 km (OSD146 Fram Strait in the Greenland Sea). However, some stations located near oceanic islands such as OSD7 (Moorea - Tiahura) in French Polynesia corresponded to truly oceanic waters. Sampling sites exhibited a wide range of temperatures and salinities: from −1.6 °C (OSD146) to 31.3 °C (OSD39 off Charleston USA), from freshwater (only one station, OSD10 in Lake Erie with 0.14 PSU) and brackish waters (for example OSD35 in Chesapeake Bay with 8.9 PSU) to marine (for example 34 PSU at OSD57, off Hawaii) and even hypersaline waters (max. salinity was 48 PSU at OSD130, in an icelandic fjord). Nitrate concentration ranged from below the detection limit (e.g. OSD6 and 14 in Mediterranean Sea or OSD56, 57 and 144 off Hawaii) to 11.7 µM off Helgoland in the North Sea (OSD3) with an average of 2.3 ± 3.3 µM. Phosphate concentration was on average 0.23 ± 0.23 µM ranging from 0 µM e.g. off Belize in the Caribbean Sea to 1.55 µM at OSD71 (Otago in New Zealand).

### Chlorophyta contribution to photosynthetic phytoplankton in coastal waters

**Figure 2:**
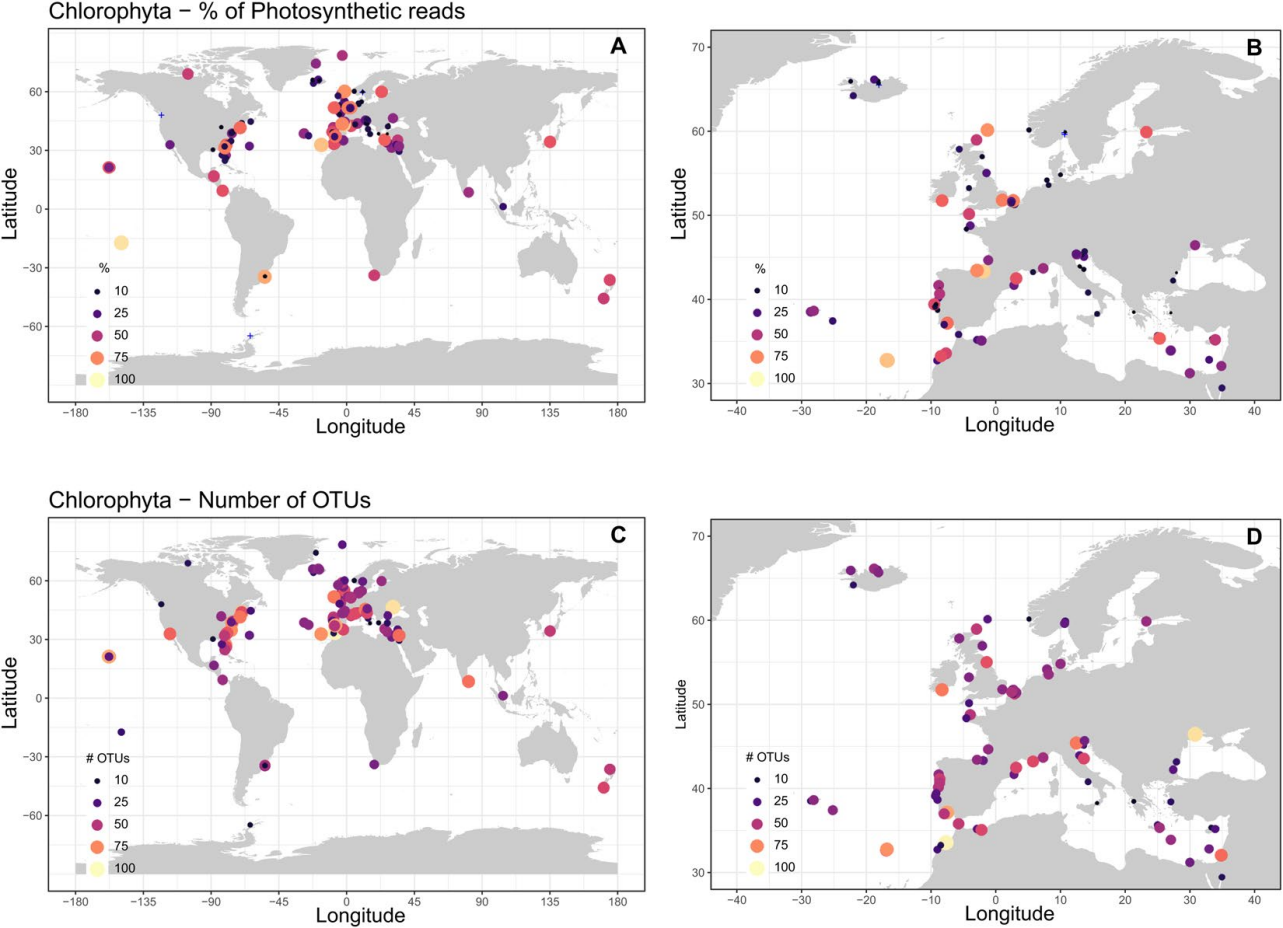
(**A**) Map of the contribution of Chlorophyta to OSD photosynthetic reads (dinoflagellates excluded). (**B**) Idem for Europe. Stations where Chlorophyta reads represented less than 1% of photosynthetic reads are represented by blue crosses. (**C**,**D**) Idem for number of Chlorophyta OTUs.

**Figure 3:**
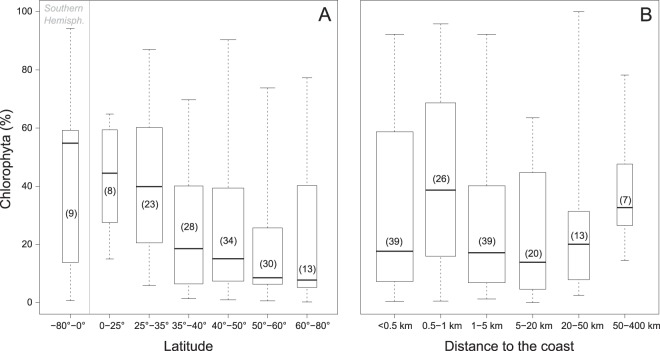
Boxplots of Chlorophyta contribution to photosynthetic reads (dinoflagellates excluded) per range of metadata. (**A**) Latitude. (**B**) Distance to the coast. Numbers in brackets correspond to the number of stations in the range (also represented by the boxplot width).

The global OSD dataset provided 1,103,675 reads of the 18S rRNA V4 regions that could be assigned to photosynthetic organisms. Dinoflagellates were excluded from photosynthetic organisms because about 50% of the species are not photosynthetic^28^ and it is difficult to precisely assign dinoflagellate OTUs to photosynthetic vs. non-photosynthetic species as no reference database with this functional information is currently available. Moreover, dinoflagellates have a large number of rRNA gene copies^29^ which causes an inflation of their representation in metabarcode studies. Chlorophyta represented 29 ± 25% of photosynthetic reads (Fig. 2A) and constituted the second most represented photosynthetic division in terms of percent of reads and number of OTUs after Ochrophyta (mostly diatoms, Fig. 1). The number of reads per station assigned to Chlorophyta ranged from 9 at OSD42 (Mediterranean Sea) to 18,570 at OSD111 (Ria de Aveiro in Portugal, Table S1) with an average of 2,217 ± 3,172 reads. Chlorophyta varied from less than 1% of photosynthetic reads at OSD41 (Alaska,), 128 (Eyafjordur 3 off Iceland), 155 (Oslo fjord off Norway), 157 (Skagerrak off Norway) and 187 (Palmer Station in Antarctica) to 94% at OSD7 (Moorea in French Polynesia). The percentage of Chlorophyta decreased from the equator (around 40% of Chlorophyta reads in average) to 60°N (circa 10%) and increased again up to 20% in high Northern latitudes (Fig. 3A). It was maximum close to the shore, between 0.5 and 1 km of the coast, decreasing in the near shore areas to increase again further away to almost 40% (Fig. 3B).

The slope of the Chlorophyta rarefaction curves was inversely proportional to the number of reads (Fig. S1) and reached saturation (slope < 0.1) for 92% of the stations. Saturation slope did not appear to be linked to the geographic origin of the samples (Fig. S1). A total of 749 OTUs (99% identity) were assigned to Chlorophyta. The number of OTUs per station was on average 38 ± 20, ranging, considering only stations with more than 100 Chlorophyta reads, from around 10 at OSD80 (off Greenland) and 174 (off Belgium) to 98 at OSD92 (off Morocco) (Fig. 2B). A weak correlation was found between the percentage of Chlorophyta and the number of OTUs at the same station (R² = 0.12, p-value = 1.3 e^−^5, data not shown).

At some stations, a high percentage of Chlorophyta corresponded to a low number of OTUs. This was the case at OSD7 (Moorea, 94%, 20 OTUs, Table S1), 50 (Bay of Biscay, 90%, 31 OTUs), 80 (Greenland Sea, 40%, 28 OTUs), 105 (Arctic Ocean, 54%, 16 OTUs) and 146 (Greenland Sea, 47%, 28 OTUs). At these stations, the Chlorophyta community was dominated by one or very few OTUs corresponding to species such as *Micromonas polaris* (OSD105 and 146) or *Carteria* sp. and *Pyramimonas* sp. (OSD80) at the high latitude stations. For OSD7, the dominant OTUs were assigned to prasinophytes clade IX and the Chloropicophyceae *Chloroparvula* sp.^7^, and at OSD50 the main OTU was assigned to an unknown Chlorodendrophyceae. In contrast, for other stations a low contribution of Chlorophyta to photosynthetic reads corresponded to a high number of OTUs: OSD22 (Gulf of Lion, 11% of Chlorophyta, 60 OTUs), OSD48 (Gulf of Venice, 4%, 33 OTUs), 72 (Baltic Sea, 6%, 42 OTUs), 95 (Singapore, 19%, 40 OTUs) and OSD178 (North Sea, 6%, 39 OTUs) (Table S1).

### Relative abundance and diversity of the different Chlorophyta classes in coastal waters

Overall, Mamiellophyceae dominated Chlorophyta in terms of mean contribution (55%, Fig. 1) and number of OTUs (304, Fig. 1). They were followed by Pyramimonadales (12%), Chlorodendrophyceae (12%), and the UTC clade (Ulvophyceae, Trebouxiophyceae and Chlorophyceae: 3.5%, 7.5% and 3.2% respectively, Fig. 1). The distribution of OTUs among the different classes was somewhat similar to the mean contribution of each class (Fig. 1). However, although Pyramimonadales and Chlorodendrophyceae had similar contribution, the former class had three time more OTUs (74) than the latter (28) (Fig. 1). Chlorodendrophyceae were dominated by OTUs with a large number of reads (29,899 reads for the larger one), while Pyramimonadales OTUs had a smaller number of reads, the larger one with 5,089 reads. Ulvophyceae and Chlorophyceae had more OTUs (respectively 95 and 94 OTUs) than expected from their relative contribution (respectively 3.5 and 3.2%). Several classes with low overall contributions had a quite large number of OTUs. For example, the Palmophyllophyceae and Pedinophyceae represented about 2 and 0.3%, respectively but had 3.4 and 1.5% of the OTUs respectively (Fig. 1). In order to estimate the level of novel diversity in each class, we computed the fraction of the OTUs with less than 98% BLAST similarity to any sequence from GenBank originating from cultures (Fig. S2). Without surprise, 100% of the OTUs from classes which have not been brought in cultures met this criterion, such as prasinophytes clade IX or VIII. In contrast, 100% of the Chloropicophyceae, despite the fact that it is a recently created class^7^, appear to match sequences from cultures suggesting that the cultivation effort has been very exhaustive for this group in coastal waters as it had been shown previously to be in oceanic waters^10^. In contrast, a large fraction of the diversity of abundant classes such as Mamiellophyceae or Pyramimonadales remains to be brought into culture.

### Distribution of specific Chlorophyta classes in coastal waters

**Figure 4:**
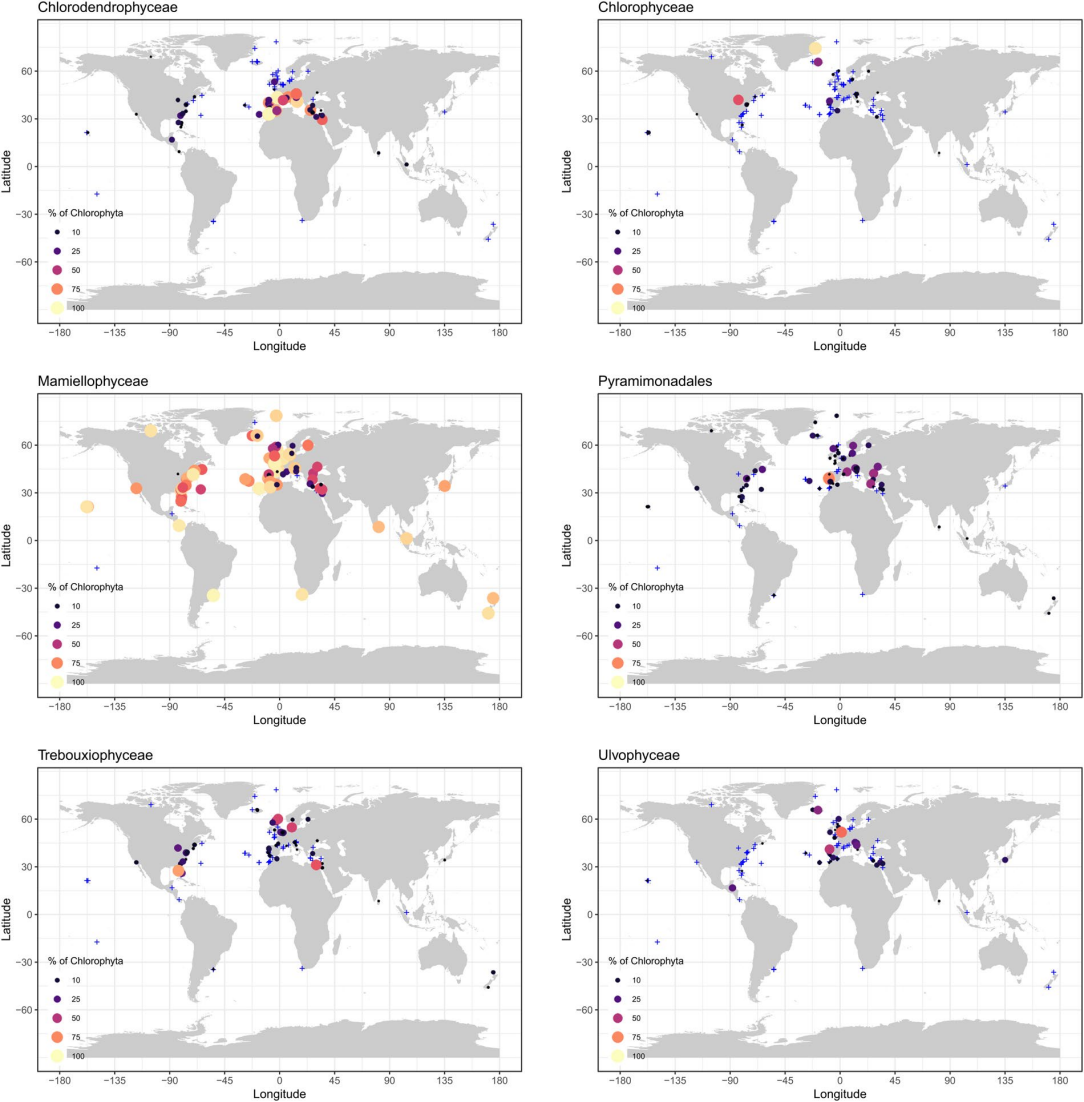
Contribution of the 6 major Chlorophyta classes at OSD stations in surface. Circle size and color are proportional to the contribution of the class relative to all Chlorophyta reads (in %). Stations where the class contributed to less than one percent of the Chlorophyta reads are represented by blue crosses. Stations with less than 100 Chlorophyta reads recorded were not considered.

**Figure 5:**
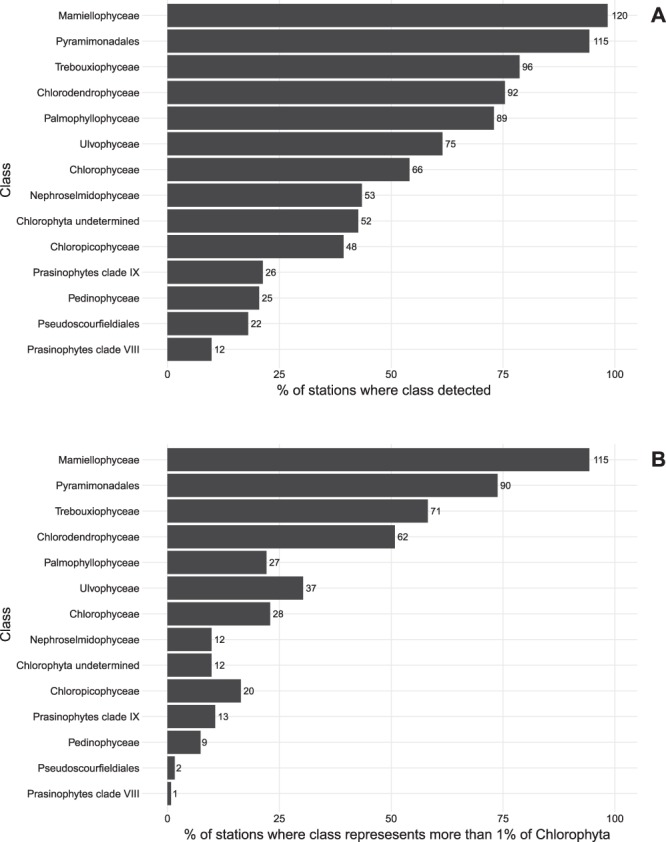
(**A**) Percentage of OSD surface stations where a given Chlorophyta class was detected (at least one read). Numbers at right of bars correspond to number of stations. (**B**) Idem but for stations where the class contributed more than 1% of the Chlorophyta reads. Stations with less than 100 Chlorophyta reads were not considered.

Mamiellophyceae were recovered at almost all stations (120 out of 122) where more than 100 Chlorophyta reads where recorded (Figs 4 and 5) and were only absent at two oligotrophic stations OSD7 and OSD28. They could reach up to 99% of Chlorophyta (OSD183 in the North Sea off Belgium). The major Mamiellophyceae OTUs (Supplementary Data S2) were assigned to the three genera *Ostreococcus* (80,988 reads), *Micromonas* (47,778 reads) and *Bathycoccus* (22,305 reads).

Pyramimonadales were also very widespread (Fig. 5) and their maximal contribution reached 90% (OSD108, Portugal coast). They were absent at some oceanic influenced stations in the Caribbean Sea (OSD28, Belize) or Atlantic Ocean, in (OSD97, Azores). No clear distribution pattern appeared for Pyramimonadales (Fig. 4). The major OTUs were assigned to genera *Pyramimonas* and *Pterosperma* (Supplementary Data S2).

Chlorodendrophyceae were less widespread than Pyramimonadales (Fig. 5), although being on average similar in relative abundance (Fig. 1). They represented up to 99% of Chlorophyta reads at OSD93 (Atlantic Ocean, off Morocco) and were abundant at Mediterranean stations (OSD4 with 91%, 6 with 58%, 14 with 81%, 24 with 82%, 94 with 43% for example, Fig. 4). Chlorodendrophyceae contribution was lower along the North American coasts (OSD28 with 16%, 41 with 3.9%, 58 with 4.6%, 60 with 12% for example, Fig. 4) and they were absent in the sub-polar North Atlantic (stations around Iceland, Greenland or Fram Strait, Fig. 4).

Trebouxiophyceae represented more than 1% of the reads at 71 stations. Their maximum contribution (80% of Chlorophyta reads) was found at OSD45 (Gulf of Mexico). They were recorded in temperate coastal waters, especially off the USA and European Atlantic coasts (Fig. 4). Trebouxiophyceae were not recorded at high latitudes nor at oligotrophic stations such as Hawaii, French Polynesia or Azores. The 3 major OTUs (7,703, 1,423 and 1,317 reads, Supplementary Data S2) were assigned to the highly diversified marine coccoid genus *Picochlorum*^30^.

Ulvophyceae maximal contribution was recorded at OSD169 (North Sea off UK, 70%). Ulvophyceae were mostly present along the North Atlantic European coast, at some stations of the Mediterranean Sea (OSD78 in the Adriatic Sea and OSD 123 off Israel, for example), in warm waterd (OSD28, 124 and 147) and in Antarctica (OSD187, Fig. 4). The most abundant Ulvophyceae OTU (Supplementary Data S2) was assigned to the macroalgal genus *Ulva*, in particular matching with 100% similarity sequences from *U. fasciata* and *U. pertusa* (synomyms of *U. australis* and *U. lactuca*, respectively), both species being considered as invasive having been carried by oysters^31^, while the second one was assigned to the marine green flagellate genus *Oltmannsiellopsis*, that is widely distributed in coastal waters^32^.

Chlorophyceae were always minor contributors to Chlorophyta and represented more than 1% of Chlorophyta reads only at 28 stations located in the Northern hemisphere (Figs 4 and 5). Their maximal contribution was reached in the Arctic Ocean (Greenland Sea, OSD80 and OSD167, 95% and 40%, respectively) and the Mediterranean Sea (OSD90, Etoliko lagoon, Greece, 57%). The major OTU (5,111 reads) was assigned to a reference sequence corresponding to *Carteria* sp. (RCC2487), a marine strain isolated from the Beaufort Sea. The second OTU (711 reads) was assigned to the very diversified genus *Chlamydomonas*, which sequences have been found in almost all ecosystems from soil to marine waters^23^. However this OTU matched at 99.7% the sequence of strain NIES-1021 which has been assigned to the marine species *Chlamydomonas kuwadae*^33^.

**Figure 6:**
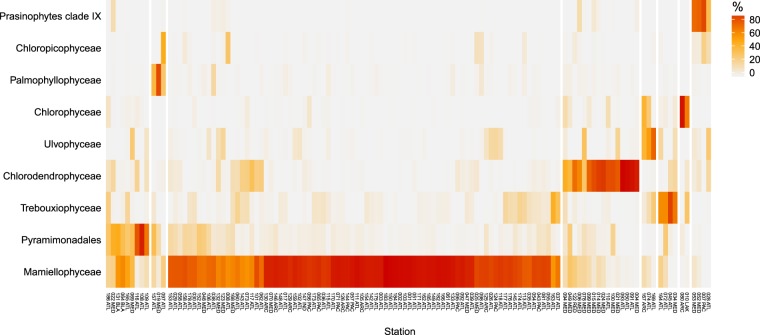
Heatmap of OSD Chlorophyta communities. Colors refer to the percentage of reads in each class related to the total number of Chlorophyta reads. Stations and classes have been clustered using Bray-Curtis dissimilarity. Stations could be split into 8 major groups (separated by white lines). Only Chlorophyta classes representing on average at least 1% of the Chlorophyta reads were taken into account. Stations with less than 100 Chlorophyta reads recorded were not considered.

The uncultivated prasinophytes clade IX represented more than 1% of the Chlorophyta reads at 13 stations mostly located in oligotrophic tropical and temperate stations (Figs 5 and S3). Their highest contributions (Figs 6 and S3) were found in the Pacific Ocean (OSD7, French Polynesia, 78%), Mediterranean Sea (OSD52 and 53, respectively 70 and 78%) and off Belize (OSD28, 34%). Major OTUs (Supplementary Data S2) were assigned to the B clade^34^.

Within Palmophyllophyceae, all OTUs were assigned to the order Prasinococcales (genera *Prasinococcus* and *Prasinoderma* for the major OTUs, Supplementary Data S2) and none to Palmophyllales, which have only be recorded from deep waters^6^. They contributed to more than 1% at 27 stations (Fig. 5), mostly in the Mediterranean Sea and along North Europe coasts (Fig. S3). Maxima were recorded off Cyprus (OSD19, 77%) and in the Skagerrak (OSD157, 37%). Interestingly they were present (but accounting for less than 1% of the reads, Fig. 5) at 62 other stations suggesting that they are probably an ubiquitous but minor component of the Chlorophyta in many environments.

Chloropicophyceae represented more than 1% at 20 stations (Fig. 5) mostly located in tropical oceanic waters. They reached their highest contribution at the Azores station OSD97 (45%) and off Bermuda (OSD8, 29%, Fig. S3). The major OTUs (Supplementary Data S2) corresponded to the species *Chloroparvula pacifica* and sp. (clades B2) and *Chloropicon roscoffensis*.

Nephroselmidophyceae represented more than 1% at 12 stations (Fig. 5) and their maximal contribution between 5 and 6% of the Chlorophyta reads were recorded in the coastal North Atlantic Ocean (OSD106 off Iceland, 152 off Canada and 157 off Norway, Fig. S3). The Nephroselmidophyceae also reached 2% at several stations in the Eastern Basin of the Mediterranean Sea (such as OSD123 off Israel, Fig. S3). The two major OTUs belonged to the genus *Nephroselmis* (Supplementary Data S2).

Pedinophyceae represented more than 1% of the Chlorophyta reads only at 9 stations (Fig. 5) and were mostly present at stations located off the USA Atlantic coast (OSD35, 46, 143, 186) and in the Mediterranean and Black Seas (OSD64 and 78, Fig. S3). The highest contribution (7.1%) was recorded in Chesapeake Bay (OSD35). The two major OTUs belonged to the genus *Marsupiomonas* (Supplementary Data S2).

The order Pseudoscourfieldiales had more than 1% of the Chlorophyta reads at only two stations (Figs 5 and S3) from the Adriatic Sea (OSD 48 and 99, 1.8% and 1%, respectively).

Prasinophytes clade VIII was the least represented group in this dataset with more than 1% at a single station (Fig. 5) off the Iberic Atlantic coast (Fig. S3).

Finally, at 13 stations (Fig. 5), more than 1% of the Chlorophyta reads could not be classified in any Chlorophyta class (Fig. S3). The maximum fraction of unclassified sequences was found in the Mediterranean Sea off Cyprus (OSD18,16%), in the Atlantic Ocean off Belize (OSD 28,8.1%) and off the East Coast of the US (OSD58, 7.5). Other unclassified reads were recovered from the Mediterranean Sea and off Iceland (OSD128).

### Chlorophyta community structure in coastal waters

Clustering based on Bray-Curtis dissimilarity defined several types of clearly defined Chlorophyta communities (Fig. 6). Some of these communities were dominated by a single class: Mamiellophyceae, Chlorodendrophyceae, Trebouxiophyceae, Chlorophyceae, Prasinophytes clade IX, Palmophyllophyceae. Among these, the Mamiellophyceae-dominated communities were the most widespread followed by the Chlorodendrophyceae-dominated communities. In contrast, some other classes such as the Pyramimonadales, Chloropicophyceae or Ulvophyceae seemed always to occur with another class, e.g. Pyramimonadales with Mamiellophyceae. Stations sampled in oligotrophic waters were dominated by prasinophytes clade IX (OSD7, 28, 52 and 53) or Chloropicophyceae (OSD 97) and these two groups rarely co-occurred (Fig. 6).

### Relationships with environmental parameters

**Figure 7:**
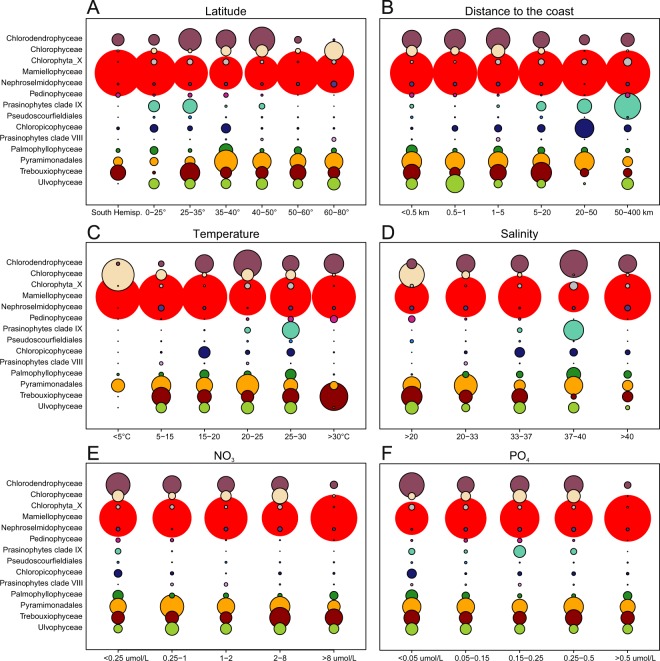
Contribution of Chlorophyta classes per range of metadata. (**A**) Latitude (OSD metadata). (**B**) Distance to the coast (calculated). (**C**) Water temperature (measured *in situ*). (**D**) Salinity (measured *in situ*). (**E**) Nitrates (World Ocean database 2013). (**F**) Phosphates (World Ocean database 2013). Circles are proportional to the average contribution of a given class to total Chlorophyta. For salinity, OSD10 was not taken into account since it is located in a freshwater lake.

Mamiellophyceae did not seem to have any marked preference with respect to the environmental parameters at the OSD stations (Fig. 7), except that they seemed to be less dominant at salinities between 37 and 40 PSU, typical of the Mediterranean Sea. The contribution of Pyramimonadales and Ulvophyceae was also similar under most environmental conditions. In contrast, some groups had marked preferences. For example, Chlorophyceae and Chlorodendrophyceae were bigger contributors at low NO_3_ and PO_4_ and close to the coast but the former were contributing more at low temperature and low salinity while it was the opposite for the latter. Two groups were typically found in oligotrophic oceanic waters, Chloropicophyceae and clade IX, as reflected by their preference for high salinity, very low nutrients (NO_3_) and large distances from the coast. However, Chloropicophyceae extended a bit more towards the coast and had a slightly wider range of temperature, compared to clade IX which was mostly found in waters between 25 °C and 30 °C. Similarly, Pedinophyceae were mostly observed in low nitrate waters above 15 °C but in contrast to the two previous groups, they could be found very close to the coast.

## Discussion

Green algae (Chlorophyta) are clearly significant photosynthetic contributors in coastal waters as demonstrated by the OSD dataset where they constituted the second major photosynthetic group (dinoflagellates excluded) after Ochrophyta (mostly diatoms) both in terms of read contribution and number of OTUs (Fig. 1). The importance of Chlorophyta had already been highlighted previously in some specific environments. In European coastal waters, the contribution of Chlorophyta to photosynthetic 18S rRNA clones was found to be 42%^35^. In the English Channel and North Sea, from 85% to 47% of the picoeukaryote cells hybridized by TSA-FISH were Chlorophyta, more precisely Mamiellophyceae^36–38^. Similar contributions were also observed in another OSD dataset^39^ focusing on a smaller number of stations and using both the V4 and the V9 18S rRNA regions (26% and 20%, respectively). In comparison, Chlorophyta have a lower overall contribution (13% in average) in the Tara *Ocean* V9 dataset from oceanic waters^10^. The number of Chlorophyta OTUs (745 at 99% similarity) was of the same order than found in other studies: in European coastal waters^40^, 314 V4 OTUs were found at 97% similarity or in the Tara *Ocean* dataset^12^, 1420 V9 OTUs were found with the SWARM algorithm which uses natural clustering rather a fixed similarity level^41^.

In the OSD dataset, the percentage of Chlorophyta was maximum in tropical waters with oceanic characteristics (94% OSD7 off Moorea, Fig. 3). Such high Chlorophyta contribution in oceanic waters have been also been observed in clone library studies^34^ as well as in the Tara *Ocean* dataset^10^. In contrast, low Chlorophyta contribution (less than 1% Chlorophyta reads) was observed at very few stations in the North Atlantic Ocean (e.g. off Norway OSD155 and 157) and in the Arctic (OSD128). Such low contribution of Chlorophyta does not mean that their abundance is always low in these waters since sampling was restricted to a single day and Chlorophyta have been previously isolated in these environments, e.g. in Norwegian coastal waters^42^.

In the OSD dataset, Mamiellophyceae (especially *Micromonas*, *Ostreococcus* and *Bathycoccus*) was the major Chlorophyta class in coastal waters under a wide range of environmental conditions, as previously reported by many studies in coastal and nutrient-rich environments from the Arctic Ocean to the Mediterranean Sea through the Pacific and Indian Oceans^36,37,43–47^. Not *et al*.^48^ found *Micromonas* to be the most prevalent genus in the world ocean coastal waters and at a more local scale, *Micromonas* dominates coastal picoplankton in the Western English Channel^37^. Collado-Fabri *et al*.^49^ and Rii *et al*.^11^ found that Mamiellophyceae (*Micromonas*, *Ostreococcus* and *Bathycoccus* mostly) were dominant in the upwelling-influenced coastal waters off Chile. Using quantitative PCR, Marie *et al*.^44^ found *Bathycoccus* to be dominant in a transect through the Mediterranean Sea.

In contrast, the contribution of Mamiellophyceae was low at oceanic OSD stations, which confirms data from the oceanic Tara *Ocean* dataset, where only 17% of the Chlorophyta reads belonged to Mamiellophyceae^10^. Nutrient depleted environments have been previously reported to host Chloropicophyceae^10^ and clade IX^11,50,51^. These two groups however appear to be differentially distributed in the OSD dataset (Fig. 6) with prasinophytes clade IX in more oligotrophic areas than Chloropicophyceae, as observed previously in the South China Sea^51^ or the Pacific gyre^34^. Picocystophyceae (formerly prasinophytes clade VIIC^7^) were completely absent from the OSD dataset, confirming that this class is absent from marine waters^10^.

Pyramimonadales were recovered everywhere in OSD and were the second most abundant Chlorophyta class as found in the Tara *Oceans* dataset^12^ and often co-occurred with Mamiellophyceae (Fig. 6). They were particularly prevalent in the Mediterranean Sea and the North Atlantic Ocean, where microplankton microscopy inventories previously recorded the presence of the genera *Halosphaera* and *Pterosperma*^52–54^. In the OSD dataset, Pyramimonadales did not show any environmental preferendum supporting the observation made by Viprey *et al*.^5^ that Pyramimonadales were found in almost all metadata ranges they sampled in the Mediterranean Sea. Pyramimonadales strains have been isolated from a large range of environments including polar^55,56^, Mediterranean^57^ and various coastal waters^58^. Surprisingly, Pyramimonadales were not recovered (Fig. 4) from coastal waters of Japan (OSD124), while numerous strain or natural samples sequences from GenBank originate from this area^23,59^, and South Africa, where a wide diversity of *Pyramimonas* have been isolated^60^.

In the OSD dataset, Chlorodendrophyceae replaced Mamiellophyceae at some stations in particular in the Mediterranean Sea and contributed to Chlorophyta off the US coast and in the Indian Ocean. In contrast they were mostly absent from boreal waters (Fig. 4). This group has been somewhat overlooked in 18S rRNA surveys, most of which focused on the picophytoplankton size fraction^37,48,51,61,62^, while Chlorodendrophyceae species, such as those from the genus *Tetraselmis*, are rather nanoplanktonic^23^. Some 18S rRNA sequences have been retrieved from the Mediterranean Sea from surface, low nutrients samples^4,5^, which corroborate the pattern observed in the OSD data. The major Chlorodendrophyceae genus *Tetraselmis* has been reported in several microscopic inventories in the Mediterranean Sea^54,63^ and North Atlantic Ocean^64,65^ and strains have been isolated in a wide range of environments^66^. Interestingly, *Tetraselmis* strains are used for biotechnology applications and can grow heterotrophically^67^, which may explain their presence in low nutrient environments (Fig. 7).

At some other OSD stations, classes from the UTC clade dominated the Chlorophyta communities. Chlorophyceae showed clear environmental preferences for low salinity and low temperature waters in this dataset. Some Chlorophyceae such as *Dunaliella* have been shown to be tolerant to a large salinity range from freshwater to marine water^68,69^ and have been recorded in coastal Arctic, Southern Ocean and Northern Europe samples^23,64,65,70^. In contrast, Trebouxiophyceae and Ulvophycae did not show environmental preferences in the OSD dataset. Some Ulvophyceae OTUs corresponded to macroalgae from the wide-spread genus *Ulva*. These OTUs could have originated from unicellular stages (gametes or zoospores) since these stages can range from 5 to 15 µm^71^ and can survive for almost one day in the water^72^ or from fragmented macroalgal thalli, since the sampling was done without prefiltration. The genus *Picochlorum* to which the major OTUs belonged consists of five “*Nannochloris*-like” species isolated from saline and marine water^30^. This genus has been reported to acclimate to a wide range of salinities and to be well adapted to saline ponds and lagoons^73,74^.

## Conclusion

The OSD dataset has some clear limitations. It corresponds to a snapshot in time and is mostly limited to surface waters near the coast. It is strongly biased towards the Northern hemisphere and very few environmental metadata are available. Still it offers an opportunity to gain insights into the contribution and distribution of Chlorophyta classes in marine coastal waters. It highlights that Chlorophyta can be the main photosynthetic group in some ecosystems. In most cases (Fig. 6), a single Chlorophyta class dominates at any given site. This work has confirmed that the Mamiellophyceae are the dominant group in coastal waters, being present at nearly all the stations (Fig. 5). One unexpected finding is that Chlorodendrophyceae can replace Mamiellophyceae as the dominant group in particular in the Mediterranean Sea. Although oligotrophic waters have been little sampled during OSD, this work confirms the importance of Chloropicophyceae and prasinophytes clade IX in these waters. Finally, while for some groups we seem to have brought almost all of the environmental diversity in culture (e.g. Chloropicophyceae), this not yet the case for widespread groups such as the Mamiellophyceae (Fig. S2), emphasizing the necessity to continue isolation work.

## Electronic supplementary material

Supplementary information

## Data Availability

Supplementary Data S1–S3 (mothur script used to process the data, OTU fasta file, Excel file with the OTU taxonomic assignation, OTU read abundance at each station along with OSD metadata), R script to produce some of the figures, as well as Supplementary information have been deposited to Figshare at 10.6084/m9.figshare.6794585.
