# Supplementary material for: Green microalgae in marine coastal waters: The Ocean Sampling Day (OSD) dataset
Source: Sci Rep. 2018 Sep 19;8:14020. doi: 10.1038/s41598-018-32338-w (PMC6145878; doi:10.1038/s41598-018-32338-w)
Supplement: Supplementary file 1 — Supplementary information [file 41598_2018_32338_MOESM1_ESM.pdf]

Green microalgae in marine coastal waters:
The Ocean Sampling Day (OSD) dataset

Supplementary Information

Margot Tragin[1], Daniel Vaulot[1, 2]

[1] Sorbonne Université, CNRS, UMR 7144, Station Biologique, Place Georges Teissier, 29680 Roscoff, France

[2] Corresponding author (ORCID 0000-0002-0717-5685): vaulot@sb-roscoff.fr / vaulot@gmail.com

July 22, 2018

Revised version: SREP-18-08305

Submitted to: Scientific Reports, July 22, 2018

These supplementary data are deposited on Figshare at https://doi.org/10.6084/m9.figshare.6794585

Supplementary Table

Table S1: OSD stations. Number of photosynthetic and Chlorophyta reads. Fraction of Chlorophyta reads. Number of Chlorophyta OTUs. Rows in light grey correspond to stations where less than 100 reads of Chlorophyta were recorded.

OSD sample code	OSD station	Stations	Ocean	Regional Sea	Country	Total number of reads	Photosynthetic reads	Chlorophyta reads	Chlorophyta %	Chlorophyta OTUs
OSD1	1	Plymouth - L4	Atlantic Ocean	English Channel	UK	27254	2 450	1 403	57.3	23
OSD2	2	Roscoff - SOMLIT	Atlantic Ocean	English Channel	France	30774	9 631	2 660	27.6	50
OSD3	3	Helgoland	Atlantic Ocean	North Sea	Germany	29094	14 537	1 224	8.4	40
OSD4	4	LTER-MC	Mediterranean Sea	Tyrrhenian Sea	Italy	28838	4 590	605	13.2	16
OSD5	5	Crete	Mediterranean Sea	Aegean Sea	Greece	17367	458	92	20.1	21
OSD6	6	Blanes	Mediterranean Sea	Balearic Sea	Spain	18660	1 512	487	32.2	31
OSD7	7	Moorea - Tiahura	Pacific Ocean	North Pacific Ocean	Tahiti	13617	1 849	1 743	94.3	20
OSD8	8	BATS	Atlantic Ocean	North Atlantic Ocean	Bermuda	13129	909	270	29.7	31
OSD10	10	Lake Erie W4			USA	47593	9 748	513	5.3	41
OSD13	13	Varna Bay	Black Sea		Bulgaria	24864	5 593	76	1.4	19
OSD14	14	Banyuls	Mediterranean Sea	Western Basin	France	25523	8 386	5 234	62.4	59
OSD15	15	Villefranche - SOMLIT	Mediterranean Sea	Ligurian Sea	France	26510	1 791	781	43.6	45
OSD17	17	VLIZ	Atlantic Ocean	North Sea	Belgium	22082	7 880	201	2.6	23
OSD18	18	Kyrenia	Mediterranean Sea	Eastern Basin	Turkish Republic of Northern Cyprus	13884	725	33	4.6	11
OSD19	19	Famagusta	Mediterranean Sea	Eastern Basin	Turkish Republic of Northern Cyprus	12403	768	402	52.3	25
OSD20	20	Faxafloi	Atlantic Ocean	North Atlantic Ocean	Iceland	8484	285	50	17.5	17
OSD21	21	Croatia	Mediterranean Sea	Adriatic Sea	Croatia	11332	530	138	26.0	12
OSD22	22	Marseille Solemio SOMLIT	Mediterranean Sea	Gulf of Lion	France	39017	4 816	553	11.5	60
OSD24	24	Marchica	Mediterranean Sea	Alboran Sea	Morocco	8516	2 770	644	23.2	27
OSD25	25	Saidia Rocher	Mediterranean Sea	Alboran Sea	Morocco	17902	368	153	41.6	20
OSD26	26	Tangier	Atlantic Ocean	Strait of Gibraltar	Morocco	18976	8 509	1 530	18.0	53
OSD28	28	Belize	Atlantic Ocean	Caribbean Sea	Belize	6467	505	288	57.0	37
OSD29	29	Florida	Atlantic Ocean	North Atlantic Ocean	USA	17630	2 935	1 239	42.2	74
OSD30	30	Tvärminne	Atlantic Ocean	Gulf of Finland	Finland	36294	2 948	1 892	64.2	43
OSD34	34	Alexandria	Mediterranean Sea	Levantine Basin	Egypt	10019	5 227	2 085	39.9	33
OSD35	35	Cheasapeake Bay	Atlantic Ocean	Chesapeake Bay	USA	28156	9 889	3 818	38.6	78
OSD36	36	Delaware	Atlantic Ocean	Chesapeake Bay	USA	23078	14 733	1 554	10.5	47
OSD37	37	Port Everglades	Atlantic Ocean	North Atlantic Ocean	USA	33922	21 490	2 769	12.9	60

OSD sample code	OSD station	Stations	Ocean	Regional Sea	Country	Total number of reads	Photosynthetic reads	Chlorophyta reads	Chlorophyta %	Chlorophyta OTUs
OSD38	38	Long Key	Atlantic Ocean	North Atlantic Ocean	USA	21069	2 461	369	15.0	50
OSD39	39	Charleston Harbor	Atlantic Ocean	North Atlantic Ocean	USA	10629	9 231	6 249	67.7	62
OSD41	41	Sequim Bay Park	Pacific Ocean	Detroit Juan de Fuca	Alaska	14137	5 290	51	1.0	12
OSD42	42	Faro Lake	Mediterranean Sea	Tyrrhenian Sea	Italy	17551	81	9	11.1	4
OSD43	43	SIO Pier	Pacific Ocean	North Pacific Ocean	USA	15928	3 371	1 073	31.8	67
OSD45	45	Tampa Bay	Atlantic Ocean	Gulf of Mexico	USA	16446	7 197	1 201	16.7	25
OSD46	46	Horn Island	Atlantic Ocean	Gulf of Mexico	USA	1954	1 493	88	5.9	10
OSD47	47	Venice Lagoon	Mediterranean Sea	Adriatic Sea	Italy	32681	26 538	4 465	16.8	44
OSD48	48	Venice Gulf	Mediterranean Sea	Adriatic Sea	Italy	13196	2 457	100	4.1	33
OSD49	49	Vida	Mediterranean Sea	Adriatic Sea	Slovenia	23505	3 075	345	11.2	31
OSD50	50	Pasaia	Atlantic Ocean	Bay of Biscay	Spain	18701	9 050	8 178	90.4	31
OSD51	51	Bocas del Toro	Atlantic Ocean	Caribbean Sea	USA	16427	4 740	2 928	61.8	44
OSD52	52	Abu Hashish	Mediterranean Sea	Levantine Basin	Egypt	12100	1 234	527	42.7	34
OSD53	53	Ras Disha	Mediterranean Sea	Levantine Basin	Egypt	10108	3 022	935	30.9	43
OSD54	54	Maine Booth Bay	Atlantic Ocean	North Atlantic Ocean	USA	27803	15 397	2 017	13.1	85
OSD55	55	Maine Damariscotta River	Atlantic Ocean	North Atlantic Ocean	USA	24687	14 876	1 742	11.7	61
OSD56	56	Hawaii Kakaako	Pacific Ocean	North Pacific Ocean	Hawaii	23061	13 937	6 195	44.5	51
OSD57	57	Hawaii Oahu	Pacific Ocean	North Pacific Ocean	Hawaii	24495	10 382	6 731	64.8	82
OSD58	58	PICO	Atlantic Ocean	North Atlantic Ocean	USA	39793	23 151	3 264	14.1	76
OSD60	60	South Carolina 2 - North Inlet	Atlantic Ocean	North Atlantic Ocean	USA	30716	16 156	2 943	18.2	64
OSD61	61	Vineyard Sound	Atlantic Ocean	Buzzards Bay	USA	22621	9 409	6 474	68.8	71
OSD62	62	Manai Straits	Atlantic Ocean	Irish Sea	UK	27570	6 101	692	11.3	37
OSD63	63	Venice Acqua Alta	Mediterranean Sea	Adriatic Sea	Italy	1545	48	18	37.5	5
OSD64	64	Odessa	Black Sea		Ukraine	41020	7 514	3 054	40.6	94
OSD65	65	Leigh Marine Laboratory	Pacific Ocean	Hauraki Gulf	New Zealand	21887	10 958	6 434	58.7	52
OSD69	69	Marghera	Mediterranean Sea	Adriatic Sea	Italy	33493	23 153	3 521	15.2	44
OSD70	70	Lido	Mediterranean Sea	Adriatic Sea	Italy	33896	11 061	3 057	27.6	72
OSD71	71	Otago	Pacific Ocean	South Pacific Ocean	New Zealand	38536	15 003	8 174	54.5	58
OSD72	72	Boknis Eck	Atlantic Ocean	Baltic Sea	Germany	16189	5 940	374	6.3	42
OSD73	73	Lima Estuary	Atlantic Ocean	North Atlantic Ocean	Portugal	17020	2 248	1 029	45.8	41
OSD74	74	Douro Estuary	Atlantic Ocean	North Atlantic Ocean	Portugal	39858	25 158	1 174	4.7	55
OSD76	76	Foglia	Mediterranean Sea	Adriatic Sea	Italy	14794	1 975	69	3.5	28
OSD77	77	Metauro	Mediterranean Sea	Adriatic Sea	Italy	6278	1 761	48	2.7	25
OSD78	78	CONISMA	Mediterranean Sea	Adriatic Sea	Italy	30482	5 465	366	6.7	59
OSD80	80	Young Sound	Arctic Ocean	Greenland Sea	Greenland	41011	13 324	5 364	40.3	11
OSD81	81	Ria Formosa Lagoon	Atlantic Ocean	Gulf of Cadiz	Portugal	14154	10 662	1 047	9.8	37
OSD90	90	Etoliko Lagoon	Mediterranean Sea	Ionian Sea	Greece	48220	2 563	59	2.3	7

OSD sample code	OSD station	Stations	Ocean	Regional Sea	Country	Total number of reads	Photosynthetic reads	Chlorophyta reads	Chlorophyta %	Chlorophyta OTUs
OSD91	91	Oualidiya	Atlantic Ocean	North Atlantic Ocean	Morocco	26139	6 970	1 650	23.7	22
OSD92	92	Casablanca	Atlantic Ocean	North Atlantic Ocean	Morocco	29465	21 589	12 478	57.8	98
OSD93	93	Eljadida	Atlantic Ocean	North Atlantic Ocean	Morocco	14626	7 743	4 783	61.8	15
OSD94	94	Saidia Marina	Mediterranean Sea	Alboran Sea	Morocco	14558	3 314	1 145	34.6	59
OSD95	95	Singapore Indigo_V	Pacific Ocean	Singapore Strait	Singapore	47718	11 320	2 141	18.9	37
OSD96	96	Sao Miguel Azores I	Atlantic Ocean	North Atlantic Ocean	Portugal	7327	455	29	6.4	6
OSD97	97	Faial Azores	Atlantic Ocean	North Atlantic Ocean	Portugal	12153	632	228	36.1	18
OSD98	98	Sao Jorge Azores	Atlantic Ocean	North Atlantic Ocean	Portugal	26906	3 136	1 319	42.1	40
OSD99	99	C1	Mediterranean Sea	Adriatic Sea	Italy	40530	2 063	298	14.4	34
OSD100	100	Crete - GOS	Mediterranean Sea	Aegean Sea	Greece	34799	4 870	3 240	66.5	45
OSD101	101	Quinta do Lorde	Atlantic Ocean	North Atlantic Ocean	Portugal	14734	8 185	7 123	87.0	54
OSD102	102	Marina do Funchal	Atlantic Ocean	North Atlantic Ocean	Portugal	43408	18 687	10 960	58.7	65
OSD103	103	Porto da Cruz	Atlantic Ocean	North Atlantic Ocean	Portugal	21988	10 163	8 843	87.0	77
OSD105	105	Cambridge Bay, Nunavut	Arctic Ocean	Coronation Gulf	USA	12327	861	467	54.2	16
OSD106	106	REYKIS	Atlantic Ocean	Ísafjarðardjúp	Iceland	15861	5 879	342	5.8	36
OSD107	107	Lisboa	Atlantic Ocean	North Atlantic Ocean	Portugal	13744	1 735	40	2.3	21
OSD108	108	Alcochete	Atlantic Ocean	North Atlantic Ocean	Portugal	22112	4 803	310	6.5	16
OSD109	109	Rosario	Atlantic Ocean	North Atlantic Ocean	Portugal	18782	3 352	214	6.4	25
OSD110	110	Figueira da Foz	Atlantic Ocean	North Atlantic Ocean	Portugal	16414	7 270	1 478	20.3	47
OSD111	111	Ria de Aveiro_1	Atlantic Ocean	North Atlantic Ocean	Portugal	42178	37 249	18 569	49.9	50
OSD114	114	BerlengasWatch	Atlantic Ocean	North Atlantic Ocean	Portugal	2377	20	12	60.0	5
OSD115	115	Santa Cruz	Atlantic Ocean	North Atlantic Ocean	Portugal	8824	2 707	308	11.4	26
OSD116	116	Lagoa de Obidos	Atlantic Ocean	North Atlantic Ocean	Portugal	19200	11 526	668	5.8	27
OSD117	117	Tavira Beach	Atlantic Ocean	Gulf of Cadiz	Portugal	29026	6 934	4 837	69.8	81
OSD118	118	Lough Hyne	Atlantic Ocean	Celtic Sea	Ireland	31394	12 052	8 062	66.9	72
OSD122	122	Station A Gulf Of Eilat	Red Sea	Gulf of Eilat	Israel	20964	1 072	183	17.1	15
OSD123	123	Shikmona	Mediterranean Sea	Eastern Basin	Israel	15955	718	164	22.8	33
OSD124	124	Osaka Bay	Pacific Ocean	Japan Sea	Japan	28429	11 412	7 207	63.2	53
OSD125	125	Cullercoats Beach	Arctic Ocean	North Sea	UK	21329	8 894	2 284	25.7	62
OSD126	126	Eyafjordur_1	Arctic Ocean	Greenland Sea	Iceland	30483	5 470	284	5.2	24
OSD128	128	Eyafjordur_3	Arctic Ocean	Greenland Sea	Iceland	36511	16 057	34	0.2	10
OSD130	130	Eyafjordur_5	Arctic Ocean	Greenland Sea	Iceland	33849	6 775	1 609	23.7	43
OSD131	131	Zlatna ribka	Black Sea		Bulgaria	13248	2 676	301	11.2	28
OSD132	132	Sdot YAM	Mediterranean Sea	Eastern Basin	Israel	13174	3 304	1 324	40.1	69
OSD133	133	Robben Island	Atlantic Ocean	South Atlantic Ocean	South Africa	27165	6 952	3 812	54.8	35
OSD141	141	Raunefjorden	Atlantic Ocean	North Sea	Norway	25913	272	21	7.7	8
OSD142	142	Gray's Reef National Marine Sanctuary	Atlantic Ocean	North Atlantic Ocean	USA	6126	734	537	73.2	28
OSD143	143	Skidaway Institute of Oceanography	Atlantic Ocean	North Atlantic Ocean	USA	33922	27 114	3 751	13.8	52
OSD144	144	Maunalua Bay O'ahu	Pacific Ocean	North Pacific Ocean	USA	14029	914	330	36.1	28
OSD145	145	Blankenberge	Atlantic Ocean	North Sea	Belgium	35678	14 033	1 210	8.6	40
OSD146	146	Fram Strait	Arctic Ocean	Greenland Sea	Germany	13698	6 054	2 863	47.3	28
OSD147	147	Rajarata	Indian Ocean	Bay of Bengal	Sri Lanka	32618	9 636	4 295	44.6	70
OSD148	148	Wadden Sea	Atlantic Ocean	North Sea	Germany	14684	6 982	698	10.0	43

OSD sample code	OSD station	Stations	Ocean	Regional Sea	Country	Total number of reads	Photosynthetic reads	Chlorophyta reads	Chlorophyta %	Chlorophyta OTUs
OSD149	149	Laguna Rocha Norte	Atlantic Ocean	Laguna Rocha	Uruguay	30791	17 462	10 340	59.2	52
OSD150	150	Laguna Rocha Sur	Atlantic Ocean	Laguna Rocha	Uruguay	33739	19 853	16 225	81.7	44
OSD151	151	South Atlantic Microbial Observatory	Atlantic Ocean	South Atlantic Ocean	Uruguay	9136	847	38	4.5	10
OSD152	152	Compass Buoy Station	Atlantic Ocean	Bedford Basin	Canada	21563	1 725	257	14.9	23
OSD153	153	Faro Island	Atlantic Ocean	Gulf of Cadiz	Portugal	18526	5 804	1 121	19.3	50
OSD154	154	Arcachon-SOMLIT	Atlantic Ocean	Bay of Biscay	France	25829	10 419	4 100	39.4	42
OSD155	155	Steilene Oslofjord	Atlantic Ocean	North Sea	Norway	8049	5 092	29	0.6	8
OSD156	156	Hvaler Tisler Site	Atlantic Ocean	North Sea	Norway	4514	2 850	70	2.5	22
OSD157	157	ELLEIm2	Atlantic Ocean	Oslofjord	Norway	44012	23 524	135	0.6	30
OSD158	158	Sao Miguel Azores II	Atlantic Ocean	North Atlantic Ocean	Portugal	10757	2 181	416	19.1	40
OSD159	159	Brest-SOMLIT	Atlantic Ocean	North Atlantic Ocean	France	13901	4 575	338	7.4	23
OSD162	162	Stonehaven	Atlantic Ocean	North Sea	UK	21074	10 325	828	8.0	36
OSD163	163	Scapa	Atlantic Ocean	North Sea	UK	14448	9 439	5 007	53.0	52
OSD164	164	Scalloway	Atlantic Ocean	North Sea	UK	5567	1 322	1 022	77.3	27
OSD165	165	Loch Ewe	Atlantic Ocean	West Coast of Scotland	UK	10815	2 620	428	16.3	39
OSD166	166	Armintza	Atlantic Ocean	North Atlantic Ocean	Spain	18000	7 201	5 456	75.8	41
OSD167	167	Eyafjordur_6	Arctic Ocean	Greenland Sea	Iceland	18183	6 136	322	5.2	38
OSD168	168	IMST_izmir	Mediterranean Sea	Aegean Sea	Turkey	56061	9 500	124	1.3	17
OSD169	169	Brightlingsea Creek, Essex	Atlantic Ocean	North Sea	UK	19391	3 236	2 358	72.9	40
OSD170	170	Belgium - 130	Atlantic Ocean	North Sea	Belgium	47655	18 311	3 335	18.2	53
OSD171	171	Belgium - 230	Atlantic Ocean	North Sea	Belgium	27561	11 771	545	4.6	24
OSD173	173	Belgium - 710	Atlantic Ocean	North Sea	Belgium	36364	13 322	937	7.0	41
OSD174	174	Belgium - 780	Atlantic Ocean	North Sea	Belgium	44766	9 711	168	1.7	10
OSD175	175	ZG02	Atlantic Ocean	North Sea	Belgium	25131	3 596	274	7.6	19
OSD176	176	Belgium - 215	Atlantic Ocean	North Sea	Belgium	34978	5 388	216	4.0	20
OSD177	177	Belgium - 120	Atlantic Ocean	North Sea	Belgium	40463	3 454	237	6.9	14
OSD178	178	Belgium - 435	Atlantic Ocean	North Sea	Belgium	51421	9 379	605	6.5	39
OSD182	182	W08	Atlantic Ocean	North Sea	Belgium	25922	4 507	1 040	23.1	28
OSD183	183	W09	Atlantic Ocean	North Sea	Belgium	39849	12 218	9 018	73.8	43
OSD184	184	W10	Atlantic Ocean	North Sea	Belgium	37131	6 084	1 667	27.4	27
OSD185	185	Belgium - 421	Atlantic Ocean	North Sea	Belgium	49290	11 886	2 203	18.5	48
OSD186	186	SERC Rhode River Maryland	Atlantic Ocean	Chesapeake Bay	USA	16403	3 322	406	12.2	30
OSD187	187	Palmer station	Southern Ocean	Drake Passage	Antarctica	23229	14 363	99	0.7	9

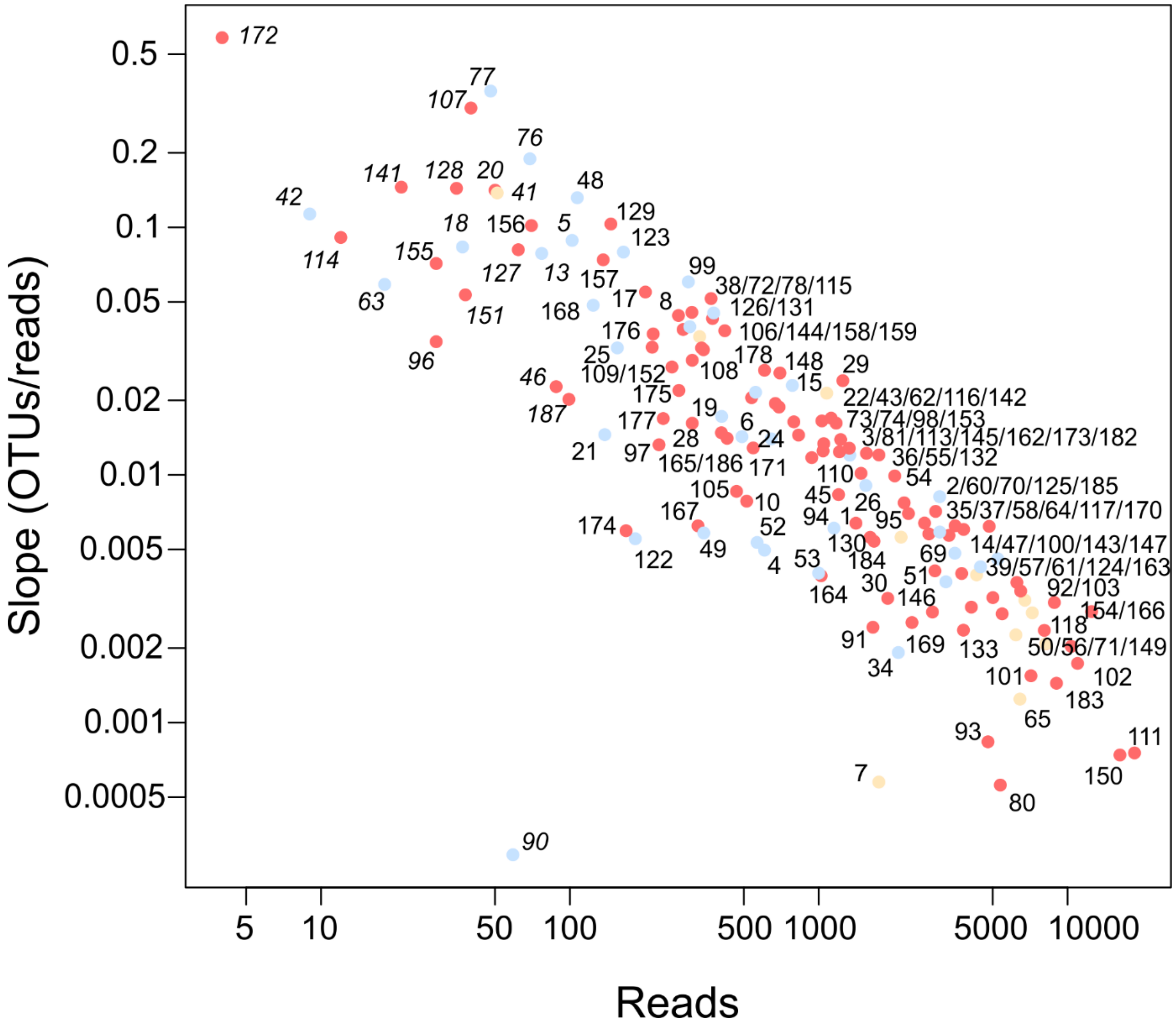

Fig. S1: Slope at the end of the rarefaction curves for Chlorophyta OTUs (99%) for OSD surface stations vs. number of Chlorophyta reads. Stations with less than 100 Chlorophyta reads (Table S1) are in italic. Color of the dots refers to the oceanic region: Atlantic Ocean (red), Pacific Ocean (ocher), Mediterranean Sea (blue).

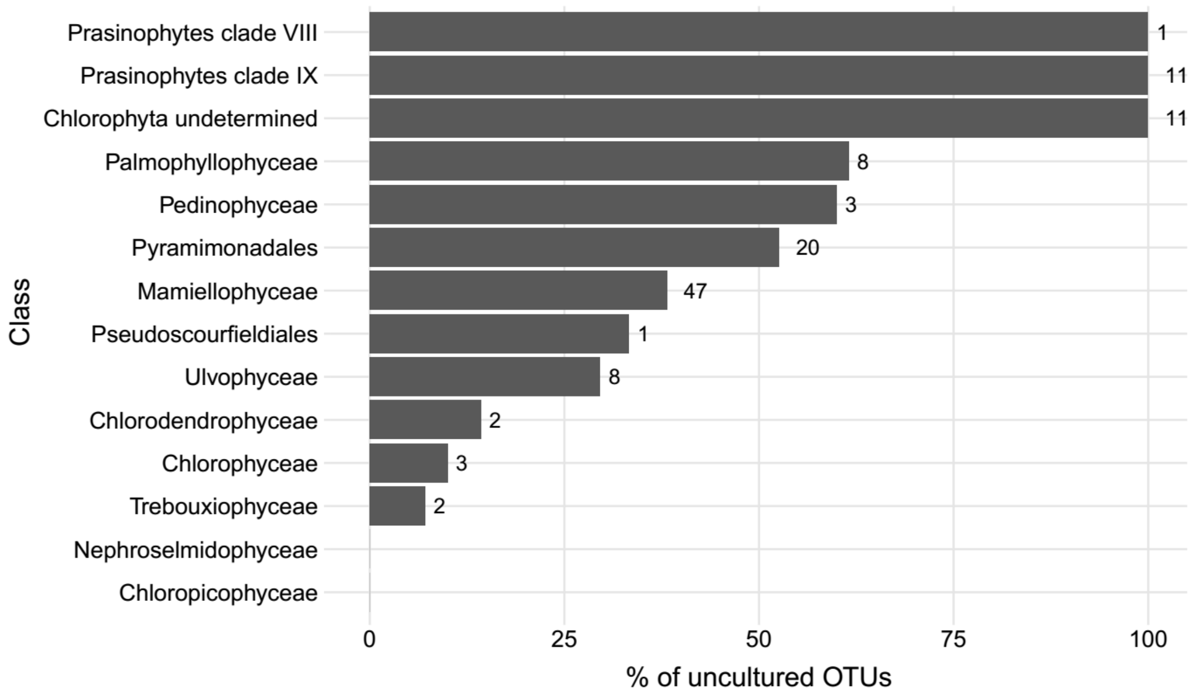

Fig. S2: Estimation of uncultured OTUs. Fraction of Chlorophyta OTUs having at least 10 reads and which do not display more than 98% sequence similarity to a Genbank sequence corresponding to a culture. Number at the right correspond to number of OTUs.

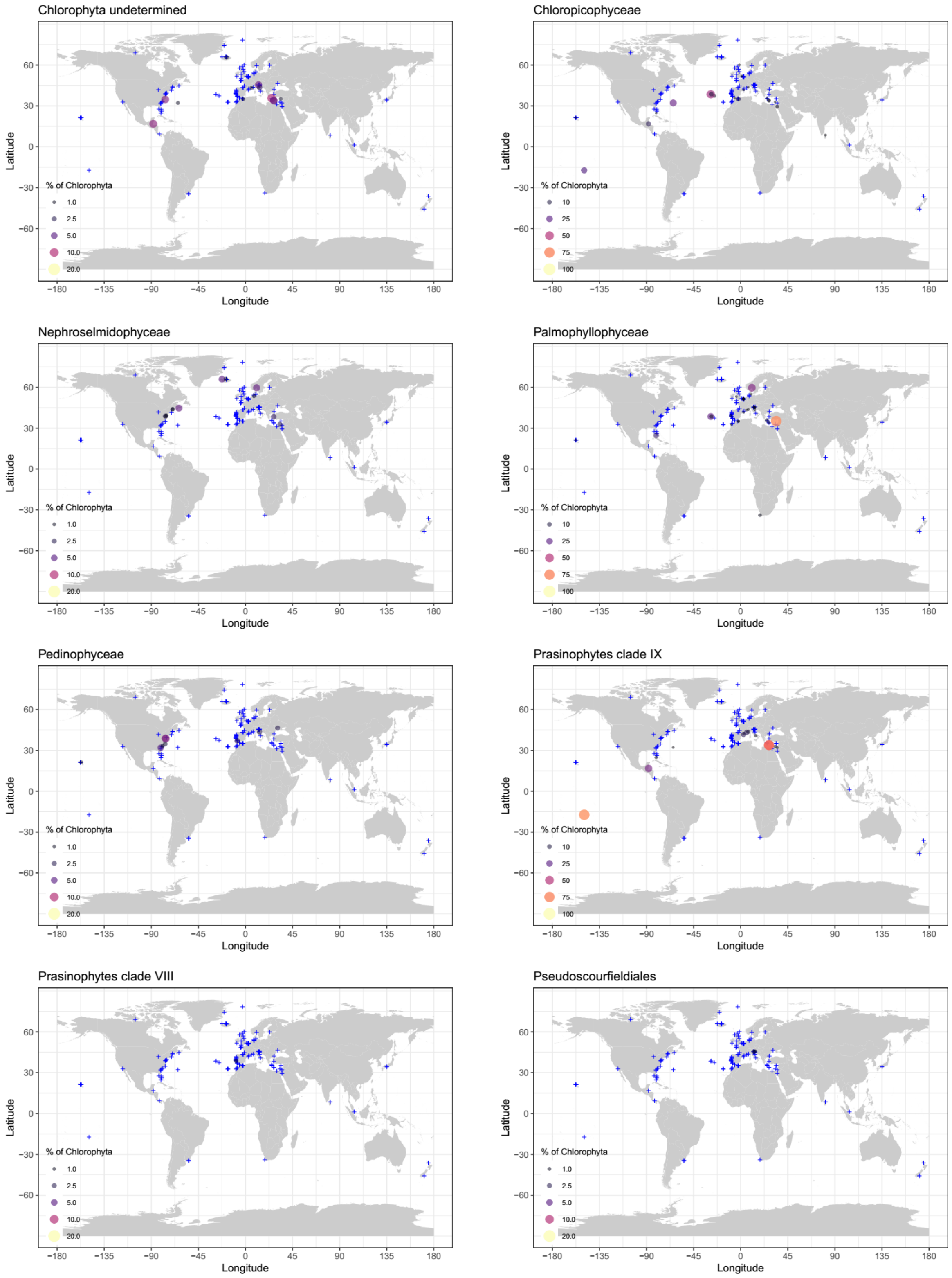

Fig. S3: Contribution of the 8 minor Chlorophyta classes at OSD stations in surface. Circle size and color is proportional to the contribution of the class relative to all Chlorophyta reads (in %). Stations where the class contributed to less than one percent of the Chlorophyta reads are represented by blue crosses. Stations with less than 100 Chlorophyta reads were not considered.

Supplementary data

Data S1: Mothur script for sequence analysis.

Data S2: Excel file. Sheet "otus": Chlorophyta OTU table with taxonomy and number of reads assigned to each OTUs per OSD 2014 samples. Sheet "samples": list of OSD samples with mention of those taken into account as originating from the water surface. Sheet "metadata": metadata for each OSD stations (see Supplementary Table 1).

Data S3: Fasta file of OSD 2014 Chlorophyta OTUs representative sequences.
